# DeWitt County Medical Society

**Published:** 1861-05

**Authors:** C. Goodbrake

**Affiliations:** Secretary


### DE WITT COUNTY MEDICAL SOCIETY.

The Society met in annual session at the office of H. Goodbrake, in Clinton, on the second day of April. The President, Dr. J. H. Tyler, in the Chair.

The minutes of the previous meeting were read and approved.

On motion of Dr. Edmiston, Dr. Edwin Wallace Gideon was invited to participate in the proceedings of this meeting.

The following officers were then elected for the ensuing year:

Z. H. Madden, M. D., President; Thos. W. Davis, M. D., Vice President; Thomas K. Edmiston, M. D., Treasurer; Christopher Goodbrake, M. D., Secretary. John Wright, M. D., J. H. Tyler, M. D., J. C. Ross, M. D., Censors.

Delegates to the Illinois State Medical Society—John Wright, M. D., B. S. Lewis, M. D., and Christopher Goodbrake, M. D.

Delegates to the American Med. Association—J. H. Tyler, M. D., and Z. H. Madden, M. D.

The President elect then took the chair, and made a few very appropriate remarks which were well received by the members of the Society.

Dr. J. H. Tyler, the retiring President, then delivered the following valedictory:

*Gentlemen of the De Witt County Medical Society:*

We have met again in annual session, for the purpose of deliberating upon and discussing those diseases which have prevailed in our respective fields of labor since our last annual meeting, and to transact such business as is prescribed by the By-Laws of this Society; such as the election of officers for the ensuing year, the appointment of essayists, and the selection of subjects for investigation.

Gentlemen, permit me to direct your attention to a few facts relative to some of the diseases which have prevailed during the past year within the circuit of our county.

Among the great variety of diseases, if there is any one which has stronger claims upon the attention of the members of this Society, than any other, it is Diphtheria—from the fact of its extensive prevalence and great fatality during the past year. Doubtless this annoying difficulty which has recently visited our region, will receive especial attention at this session of the Society.

Quite a number of cases of dysentery occurred last autumn, but fortunately most of which, under appropriate treatment, terminated favorably; some, however, were of the gravest character, and notwithstanding they received scientific and well directed management, rapidly progressed to a fatal issue.

Malarious fevers, such as have been heretofore regarded unalarming and easily cured, assumed a condition peculiarly annoying to the practitioner and dangerous to the aged and infirm—many attacks bidding defiance to the most potent remedies and ultimately destroying the patient's life. One of the most prominent and useful citizens of this town, and an officer of this county, was ushered into eternity, by one of these attacks of bilious fever, in defiance of the best medical skill our county affords. Many other cases resisted all therepeutical means that could be devised, and death was the final result. The severe character assumed by these diseases the past year, in our midst, should excite a thorough investigation of malarious diseases.

Gentlemen, we now propose to say something in regard to this Society, as it appears necessary in all cases to take a retrospective view of our past history, in order to ascertain whether we have accomplished anything worthy of merit, or deserving of censure; and then we will be able to determine with some degree of accuracy whether this organization has effected or is approximating the object for which it was instituted.

On the 8th day of March, 1856, a few physicians met in this town at the office of Drs. Goodbrake & Edmiston, and after a free interchange of opinions in regard to the benefits that would be likely to accrue to the physician as well as to the afflicted, from a Medical Association in our county, the following resolution was adopted:

"That we, the Practitioners of Medicine and Surgery of De Witt County, for the purpose of promoting harmony and good fellowship, and of elevating medical and the collateral sciences, do associate ourselves together, &c."

After which officers were elected, a Constitution and By-Laws adopted, and in order to more clearly define the duty of physicians to their patients, the public, and their professional brethren, the National Code of Ethics was adopted. Since the meeting referred to, which constitutes the starting point of this Society, six annual and fifteen quarterly sessions have been held in different portions of the county, at every one of which interesting cases have been reported, able essays read, and the pathology and treatment of diseases thoughtfully discussed and carefully considered. Accessions have also been made, from time to time, to this organization, until at present nearly every regular practitioner in the county has become a member and contributes to its interests, and derives the inestimable benefits accruing from such a connection. There are doubtless quite a number in this county who receive the appelation of Doctor, and who are attempting to palm themselves upon this community as such, and who desire to be connected with this Society, but do not possess the necessary qualifications to entitle them to membership in this Society, and are therefore without the organization from necessity and not from choice.

The good feeling and friendly intercourse contemplated in the organization have been realized to a creditable extent, and our consultations, in the main, have been conducted strictly in accordance with the National Code of Ethics. But owing to circumstances (we trust of an unavoidable character)

some of our members have, in a very perceptible degree, transcended the bounds of medical propriety. Such being the case, we trust the reclaiming powers of the Society will be manifested in an effort to extricate these members from the unpleasant dilemma in which, as we fear, they have voluntarily placed themselves.

On motion, the thanks of the Society were tendered to Dr. Tyler, and a copy of his address requested to be incorporated with the minutes of this meeting.

Dr. Tyler reported a case of Endo-Carditis.

Dr. Madden reported a case of Uterine Hœmorrhage.

Drs. Edmiston, Adams and Davis were appointed Essayists for the next meeting.

Diphtheria was chosen as the subject for discussion at the next meeting of the Society.

On motion of Dr. Wright, it was ordered that the proceedings of the meeting be published in the Central *Transcript*; also in the *Chicago Medical Journal*, and *Medical Examiner*.

On motion, the Society adjourned to meet in quarterly session, at Santa Anna, on the first Tuesday of July next.

C. GOODBRAKE, M. D., *Secretary*.

